# Young-onset colorectal cancer in the North East of Scotland: survival, clinico-pathological features and genetics

**DOI:** 10.1186/s12885-020-6606-0

**Published:** 2020-02-10

**Authors:** Sarah Perrott, Kirsten Laurie, Kirsten Laws, Annie Johnes, Zosia Miedzybrodzka, Leslie Samuel

**Affiliations:** 1 University of Aberdeen, School of Medicine, Medical Sciences, Nutrition and Dentistry, Aberdeen, Scotland; 2 NHS Tayside, Ninewells Hospital, Aberdeen, Scotland; 3 NHS Grampian, Department of Clinical Oncology at Aberdeen Royal Infirmary, Aberdeen, Scotland; 4 NHS Grampian, Clinical Genetics Service, Aberdeen Royal Infirmary, Aberdeen, Scotland

**Keywords:** Young-onset colorectal cancer, Survival, Clinicopathological characteristics, Genetic referral, Microsatellite instability

## Abstract

**Background:**

Colorectal cancer (CRC) in patients aged under 55 years is on the rise, constituting approximately 10% of cases. Our aim was to determine the survival and clinico-pathological details of young-onset CRC (yCRC), as well as audit the referral rate to genetic services and thus establish the incidence of inherited cancer syndromes.

**Methods:**

A retrospective case note review was conducted for patients aged under 55 years who were diagnosed with CRC between 2005 and 2015 in the North East of Scotland. Cases were identified by pathology records and data was obtained from patient notes. Analysis was performed using SPSS version 25 (IBM, New York, USA) to produce Kaplan-Meier survival estimates, descriptive statistics and markers predictive for genetic referral.

**Results:**

Data from 345 patients (age range 22–54 years) were identified. The one year, five year and overall survival rates were found to be 89, 63 and 55%, respectively. Most patients (61%) presented with advanced disease. Of 201 patients that met criteria for genetic referral, only 93 (46%) were referred to genetic services. Microsatellite instability (MSI) was identified in 14% of those referred.

**Conclusion:**

Survival in yCRC was found to be better than that in later onset disease, despite higher rates of advanced disease. Patients were under-referred to genetic services, where a significant proportion were found to be MSI positive and investigated for Lynch syndrome.

## Background

Traditionally a disease of the elderly, colorectal cancer (CRC) incidence in the young is steadily rising across the globe [1, 2]. In contrast, the incidence of CRC in older patients is seeing a progressive decrease in the developed world, which is likely to be attributed to population-based CRC screening [3–5]. CRC is the third most common cancer worldwide, with approximately 10% of cases affecting patients aged under 55 years [4, 6]. These younger patients often present with more advanced disease and adverse pathological features compared to their older counterparts [7]. This may have a negative impact on their survival outcome [8].

Evidence regarding young-onset CRC (yCRC) patient prognosis is conflicting. Some retrospective studies suggest that younger patients have a poorer prognosis than those with later-onset disease [8]. However, other studies suggest their prognosis is better or equivalent to those aged over 55 years [9].

Inherited predispositions to CRC are sometimes responsible for causing the disease, especially within the younger demographic [8]. These predispositions can be divided into low-penetrance familial clusterings and high-penetrance autosomal dominant cancer syndromes [3]. The former carry a low associated risk to family members and are assumed to have polygenic origin [3]. The latter are usually defined by germline mutation in mismatch repair genes in the case of Lynch syndrome or by a germline mutation in the adenomatous polyposis coli (APC) gene for familial adenomatous polyposis (FAP) [3, 8]. Other causes of CRC are deemed sporadic and are not thought to have a germline genetic predisposition. The population prevalence of Lynch syndrome has been estimated to be as high as 1:200 in some studies [10], with an associated lifetime risk of developing CRC of 50–70% as well as an increased risk of endometrial, ovarian and urothelial malignancies [11]. FAP accounts for approximately 1% of all CRC cases and carries a 100% lifetime risk of developing CRC [12]. Diagnosing Lynch syndrome or FAP – rather than sporadic CRC – has serious implications regarding a patient’s management and family prevention [12, 10].

Given the relatively limited and conflicting data regarding this expanding subgroup of yCRC patients, the primary aim of this study was to determine the survival outcomes and clinico-pathological features of CRC patients aged under 55 years in the North East of Scotland. During the time period of this study, the Scottish Intercollegiate Guidelines Network (SIGN) criteria were used by the healthcare team in Aberdeen Royal Infirmary to decide whether a patient should receive a genetic test based on their age and family history [13, 14]. Considering the growing relevance of genetic results in yCRC patients’ management, the secondary aim of this study was to determine the referral rate to genetic services and to establish the incidence of MSI in this patient subgroup.

## Methods

Patients were initially identified using pathology records held at Aberdeen Royal Infirmary (ARI). The population included patients from Aberdeen City, Aberdeenshire, Orkney, Shetland and Moray. Inclusion criteria for the study were a diagnosis of CRC between 2005 and 2015 and aged between 18 and 55 years old at the time of diagnosis. Patients with unavailable or insufficient notes and those who did not fit the pre-specified inclusion criteria were excluded from the study. One patient was lost to follow-up.

Following registration with and the approval of the study by NHS Grampian Clinical Audit Unit, data were extracted retrospectively from the NHS Grampian general and genetic patient records, using the electronic case record supplemented by paper files when required. The general records are kept for the purpose of patient care. The genetic case records are family based and facilitate implementation of screening of the patient’s relatives. De-identified data on patient characteristics (age at the time of diagnosis, year of diagnosis, sex, presentation and relevant past medical history); family history (including genetic referral, referral source and the outcome of referral); pathological details of tumour (location, TNM staging, numerical staging, tumour differentiation and genetic markers of tumour); treatment approach; genetic care (referred to genetics services, seen in clinic, undergone genetic testing and the associated result) and survival (disease recurrence, patient deaths and time to death) were extracted. Disease recurrence was defined by the presence of disease post-treatment on follow-up and imaging.

Data was analysed using SPSS statistics version 25 (IBM, New York, USA). ﻿ Kaplan-Meier estimates were used to evaluate survival and also compare survival outcomes between age groups of CRC, presentation type, chemotherapy agent, sex, tumour differentiation, numerical staging, year of diagnosis and microsatellite instability (MSI) status. The log rank test was used to evaluate results, with *p* < 0.05 deemed as significant. Descriptive statistics for age, sex, stage, site of cancer, co-morbidities and treatment received were initially expressed as median and interquartile range and percentages.

Genetic referral was assessed according to SIGN guidelines in use when the cohort began (2005). Thus, the prior guidelines from 2003 were used. The SIGN guidelines currently used in clinical practice were updated in 2011, although the differences between these and the 2003 guidelines with regard to genetic referral for CRC patients is negligible. To maintain consistency, the 2003 guidelines were used throughout this study despite being updated in 2011. This did not affect data collection. They are summarised below:

1. All patients aged under 50 years require referral to genetic services
2. All patients at a medium/high risk require referral to genetics services – see Table 1

**Table 1:** Summarised SIGN 2003 guidelines defining medium and high risk families

High Risk	A least three family members affected by CRC, or at least two affected by CRC and one affected by endometrial cancer. One relative must be aged ≤50 years and one must be a first degree relative.	Medium Risk	One first degree relative with CRC ≤45 years
	Gene carriers (HNPCC)		Two first degree relatives affected (one aged < 55 years)
	Untested first degree relatives of gene carriers		At least two relatives with CRC or endometrial cancer who are first degree relatives of each other

## Results

### Population

Between 03/01/2005 and 22/12/2015, 418 patients aged under 55 years with a primary CRC were initially identified from the NHS Grampian pathology record. In total, 73 patients were excluded from the study due to no cancer found on further investigation, aged over 55 years at diagnosis, diagnosed before 2005 or after 2015 or had unavailable or insufficient notes (30 patients). Therefore, the final number included in the study was 345. Age ranged from 22 to 54, with a mean age of 47.7 years (SD ± 6.1 years).

### Survival

**Fig. 1:**
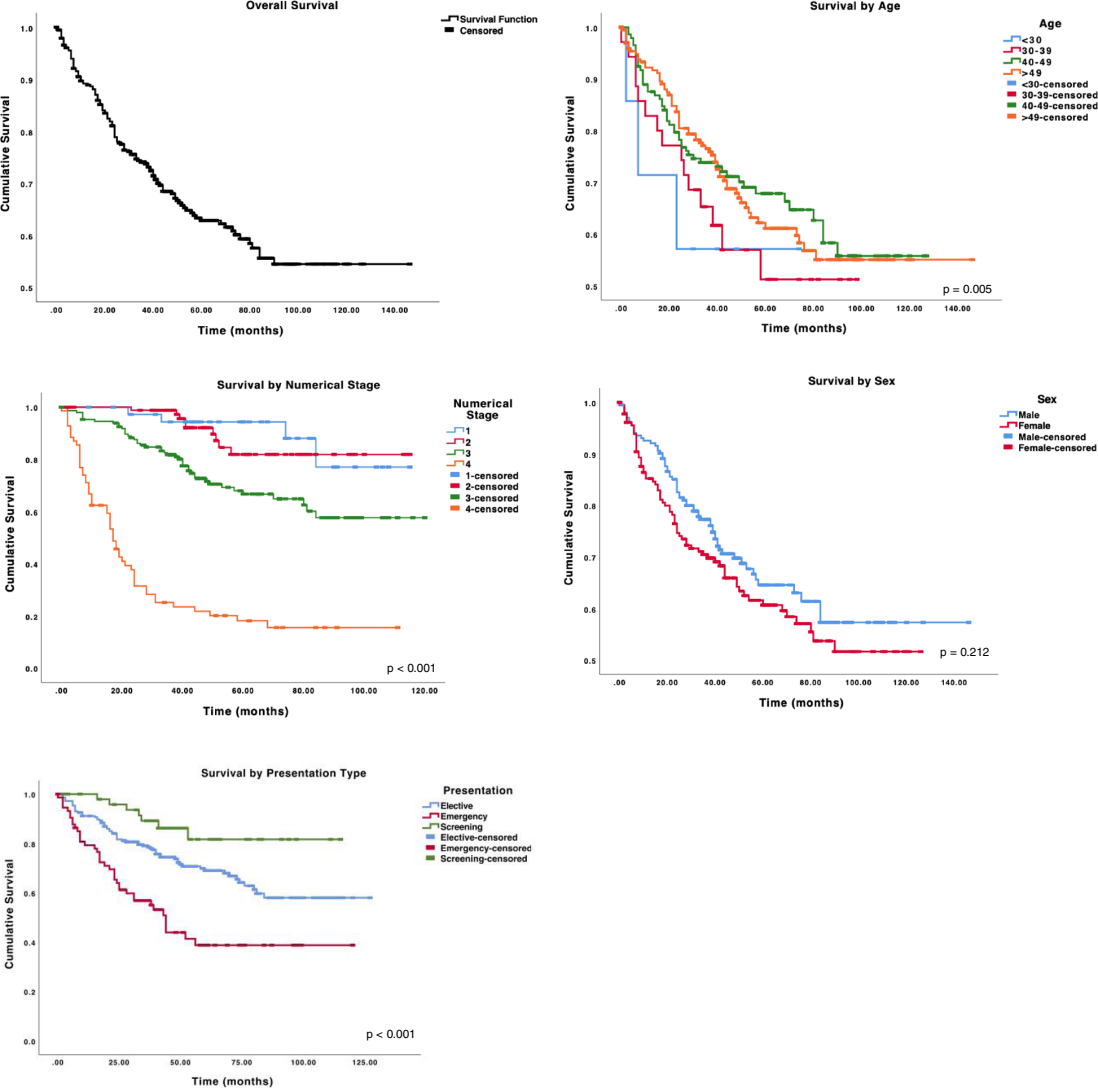
Kaplan-Meier Survival Charts

Death attributed to CRC occurred in one third of patients. Mean overall survival for CRC under 55 years was 96.1 months (95% confidence interval [CI], 89.2–102.9 months). In the univariate analysis, the following factors were associated with longer survival: increased age, stage I or II disease and presentation via screening. ﻿All patients had at least 2 years follow-up and 80.6% of the population had at least 5 years follow-up. The survival plots are shown in Fig. 1 and the corresponding survival data is detailed in Table 2. Five year survival rates in those aged less than 40 years was found to be worse (57%) compared to those aged between 40 and 54 years (68–62%).

**Table 2:** Survival Data

	Mean survival (months) ± SE	Five year survival (%)	*P*-value
Overall	96.1 ± 3.5	63	
Age group			0.005
< 30	46.9 ± 12.1	57	
30–39	62.1 ± 7.0	57	
40–49	87.3 ± 4.5	68	
50–54	96.2 ± 4.8	62	
Numerical Stage			< 0.001
I	104.0 ± 5.0	94	
II	102.4 ± 3.9	82	
III	86.8 ± 4.0	67	
IV	32.2 ± 4.6	18	
Sex			0.212
Male	99.3 ± 4.8	64	
Female	81.1 ± 4.1	61	
Presentation Type			< 0.001
Elective	90.1 ± 3.7	69	
Emergency	61.0 ± 6.3	39	
Screening	100.4 ± 5.1	82	

### Clinico-pathological characteristics

In patients with yCRC, disease presented symptomatically in 60.0%, as an emergency in 22.0% and incidentally in 0.6% of patients. Only 30.0% of the patients aged 50–55 years were identified through the bowel screening programme. A small number of patients (4.0%) had a past medical history of inflammatory bowel disease (IBD). Similarly, 4.0% had had an unrelated previous cancer such as testicular, vulval, breast or kidney cancer. One patient had had a previous CRC. The distribution of I-IV staging was 12, 26, 43 and 19%, respectively. In patients aged under 40 years, 75.0% presented with advanced disease – i.e. stage III or IV disease. This is a greater proportion compared to patients aged between 40 and 54 years where 59.3% presented with stage III or IV disease. A majority of tumours (43.1%) were located in the rectum and 3.5% of the cohort were identified to have synchronous disease. Histologically, tumour differentiation was reported as “well”, “medium” and “poor” in 1.2, 78.6 and 12.2% of patients, respectively. Patients deemed incurable at diagnosis (10.7%) did not receive surgical or endoscopic tumour resection due to late stage disease. Adjuvant or neo-adjuvant chemotherapy was administered to 81.3 and 34.1% had received radiotherapy. Cancer recurrence occurred in 28.2% of patients and 33.4% died of CRC. Epidemiological and clinicopathological characteristics of the patients by age are displayed in Table 3.

**Table 3:** Epidemiological and clinicopathological characteristics of patients by age

Characteristic	Age (n/%)				Total
	≤ 29	30–39	40–49	50–54	
Count	6 (1.7%)	34 (9.9%)	132 (38.2%)	173 (50.1%)	345 (100%)
Sex					
Male	3 (0.9%)	19 (5.5%)	69 (20.0%)	91 (26.0%)	182 (52.8%)
Female	3 (0.9%)	15 (4.3%)	63 (18.0%)	82 (23.8%)	163 (47.2%)
Presentation					
Elective	2 (0.6%)	21 (6.1%)	95 (27.5%)	89 (25.8%)	207 (60.0%)
Emergency	3 (0.9%)	12 (3.5%)	33 (9.6%)	28 (8.1%)	76 (22.0%)
Screening	0	0	1 (0.3%)	52 (15.1%)	53 (15.4%)
Incidental	0	0	1 (0.3%)	1 (0.3%)	2 (0.6%)
Unknown	1 (0.3%)	1 (0.3%)	2 (0.6%)	3 (0.9%)	7 (2.0%)
Past Medical History					
IBD	0	4 (1.2%)	3 (0.9%)	7 (2.0%)	14 (4.1%)
Previous cancer	0	1 (0.3%)	4 (1.2%)	9 (2.6%)	14 (4.1%)
Alcoholism	0	2 (0.6%)	12 (3.5%)	14 (4.1%)	28 (8.1%)
Numerical Stage					
I	0	2 (0.6%)	12 (3.5%)	27 (7.8%)	41 (11.9%)
II	2 (0.6%)	5 (14.5%)	37 (10.7%)	46 (13.3%)	90 (26.1%)
III	3 (0.9%)	16 (4.6%)	59 (17.1%)	68 (19.7%)	146 (42.3%)
IV	1 (0.3%)	10 (2.9%)	23 (6.7%)	31 (9.0%)	65 (18.8%)
Tumour Location					
Right	1 (0.3%)	6 (1.7%)	18 (5.2%)	35 (10.1%)	60 (17.4%)
Left	3 (0.9%)	12 (3.5%)	53 (15.4%)	61 (17.7%)	129 (37.4%)
Rectal	2 (0.6%)	16 (4.6%)	55 (15.9%)	74 (21.4%)	147 (42.6%)
Transverse	0	0	3 (0.9%)	2 (0.6%)	5 (1.4%)
Tumour Differentiation					
Well	0	0	2 (0.6%)	3 (0.9%)	4 (1.2%)
Medium	5 (1.4%)	23 (6.7%)	106 (30.7%)	137 (39.7%)	271 (78.6%)
Poor	1 (0.3%)	8 (2.3%)	17 (4.9%)	16 (4.6%)	42 (12.2%)
Undocumented	0	3 (0.9%)	8 (2.3%)	17 (4.9%)	28 (8.1%)
Management					
Surgical	5 (1.4%)	27 (7.8%)	117 (33.9%)	155 (44.9%)	304 (88.1%)
Palliative	1 (0.3%)	6 (1.7%)	14 (4.1%)	16 (4.6%)	37 (10.7%)
Chemotherapy Received	5 (1.4%)	31 (9.0%)	110 (31.9%)	133 (38.6%)	279 (80.9%)
Radiotherapy Received	0	14 (4.1%)	42 (12.2%)	61 (17.7%)	117 (33.9%)
Tumour Recurrence	0	5 (1.4%)	40 (11.6%)	33 (9.6%)	78 (22.6%)
Patient Deceased	3 (0.9%)	14 (4.1%)	42 (12.2%)	55 (15.9%)	114 (33.0%)

### Genetic referral

**Fig. 2:**
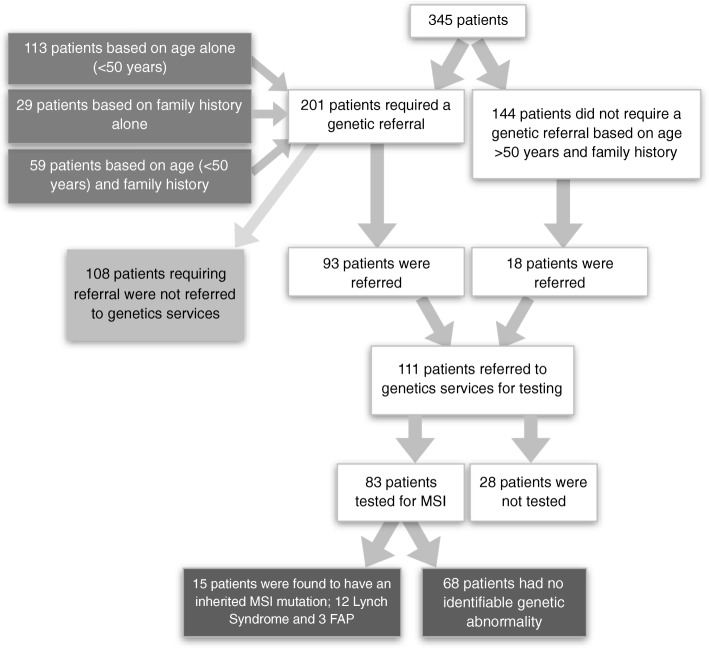
Flow-chart of Patients Referred and Tested by Genetic Services

Family history (FH) was clearly documented in the general case record notes of 185 (53.6%) patients. A positive FH for CRC was reported in 91 (26.4%) patients, of which 53 were first-degree. FH of associated Lynch syndrome tumours were also documented where possible; endometrial cancer and breast cancer FH was positive in 10 (2.9%) and 25 (7.2%) patients, respectively. SIGN guidelines (Table 1) were used to identify those requiring genetic referral. All cases aged under 50 years required referral – 172 patients (49.9%) – and those also with a first-degree FH of Lynch-related tumours – 88 patients (25.5%) – therefore qualified. Where positive FH had been documented but details of age or affected family member were unclear, cases were categorised into referral required (4 cases). In total, 201 (58.3%) of the cohort required genetic referral. Of these, 93 had such referral documented, however 108 patients who fulfilled the SIGN criteria to receive genetic testing for MSI were not referred to genetic services. An additional 18 patients were referred that did not require a genetics referral based on age or family history according to the SIGN 2003 guidelines. However, 28 of those referred did not get tested. Figure 2 demonstrates the referral of patients to genetic services. Of the 83 tested, 12 patients (14.5%) were identified to have Lynch syndrome and 3 patients (3.6%) identified to carry FAP.

As outlined in Fig. 2, a total of 111 patients (32.2% of cohort) were referred to genetic services. A further 28 were also referred to genetics but not seen; possibly due to patient choice, inability to attend appointments or patient death. After assessment by genetics services, family risk was formally categorised. The low risk category included 53 patients (47.7%), 43 patients (38.7%) were at medium risk, and 15 patients (13.5%) were at high risk. The risk was also analysed according to the original referral required category, as shown in Table 4. Four of the cases where family risk was considered high after genetic testing were originally deemed unnecessary for referral.

**Table 4:** Referral category against risk

	Categorised Risk (n)				
	Unable to determine	Low	Medium	High	Total (n)
Referral required	28	42	12	11	93
Referral not required	0	11	3	4	18
**Total**	28	53	15	15	**111**

Actual family risk of CRC according to characterisation of requirement to genetic referral

## Discussion

The incidence of CRC is increasing in young patients with seemingly few risk factors [9]. Currently, the notion that younger age of onset is related to a poor prognosis is controversial; survival data is rather ambiguous and tumour characteristics are not consistently described for this subgroup of yCRC patients [2, 8, 9]. Furthermore, no such study has been completed in Scotland. By establishing survival and characterising clinico-pathological features of this Scottish cohort, current knowledge and practice relating to yCRC may improve on both a local and international level.

In this retrospective study, survival in yCRC was found to be better than that in later onset CRC despite higher rates of advanced disease. Spanning an 11 year period up to December 2015, follow-up and survival data from 345 CRC patients aged under 55 years was collected. Despite a significant number of patients were diagnosed at late stage disease, the cohort had a greater overall 5 year survival of 63%, compared to 59% - the latest 5 year survival statistic for CRC across all age groups [15]. This may reflect the fitness and relative lack of co-morbidities of these younger patients, making them better candidates for surgery, chemotherapy and radiotherapy. We found the 5 year survival of stage III and IV patients to be 67 and 18%, respectively. Across all age groups, 5 year survival in patients stage III disease is estimated at 63%, and stage IV is 7% [15]. This suggests, contrary to popular belief, that the young-onset cohort actually had similar or better outcomes than colorectal cancer patients overall. This superior stage-specific survival is also reflected in other multi-national studies [2, 4, 9, 12]. However, in contrast, these studies did not find survival outcomes to be greater even when unadjusted to stage. This is likely to be because these studies tended to focus on patient groups aged between 20 and 40 years, rather than aged under 55 years. When our results are adjusted to age-specific survival, those less than 40 years of age were also found to have a poorer prognosis. Interestingly, one study consistent with this result by Ballester et al. also found ﻿that - despite an overall better prognosis - yCRC patients had a higher incidence of recurrence and development of metastasis than later-onset disease. In this study, tumour recurrence occurred in 22.6% of patients. In a Korean study considering CRC across all ages, recurrent disease was found to occur in 18.3% [16]. Although inconclusive, this is potentially an area for further investigation to determine why yCRC patients may have a higher incidence of tumour recurrence.

This study also characterised the cohort in terms of clinicopathological features, which found locally advanced rectosigmoid disease to be typical of yCRC. Tumours were commonly located in the left side of the bowel, with a greater proportion of tumours (42.6%) occurring in the rectum – concurring with previous literature describing young-onset disease [7]. Although our cohort is relatively small, it includes all the patients in a geographical area, including both urban & rural populations. Over 60% of patients presented with late stage (III or IV) disease. According to cancer research statistics, in Scotland approximately 23% of CRC patients present with stage IV disease and 25% with stage III disease [17]. In this cohort, perhaps surprisingly, fewer patients presented with stage IV disease (18%), although there were nearly 70% more patients (42%) diagnosed initially with stage III disease compared to the national incidence across all ages. Advanced disease prior to diagnosis may be explained by delays in patient presentation and diagnosis due to the relative rarity of the condition in comparison to the older population, as well as a lack of screening. In Scotland, population screening does not begin until the age of 50. ﻿In contrast with previous studies investigating yCRC [12, 18], incidence of poorly differentiated – or high-grade – histology was not found to be overrepresented in our patient group. An Australian study concurs with this finding, putting this discrepancy down to the subjective nature of determining tumour grade across the world [2].

Of those referred to genetic services, a significant proportion (18%) were found to be MSI positive. FH was often not documented sufficiently or, in some cases, not at all. FH is a major determining factor for referral to genetic services given the autosomal dominant inheritance pattern of Lynch syndrome. SIGN 2003 guidelines state that a ‘three generation family history should be taken from all patients with colorectal cancer’ [14]. This was not adhered to and hindered the assessment of whether a patient required genetic referral, both for the purposes of this audit and in clinical practice. The audit found that only 46% of patients categorised as requiring referral had indeed been referred to genetics as part of their cancer management. However, given the poor FH records, there were potentially more unidentified patients who required referral. This falls far below the audit standard; 54% of the unseen patients requiring referral potentially have an unidentified underlying genetic risk implicating not only their future health, but also their families. Failure to identify these high or medium risk families may have grave repercussions on the mortality and morbidity of these patients, as having knowledge of this risk allows access to the appropriate screening and counselling. Interestingly, four of the 18 patients categorised as not requiring referral (therefore were aged over 50 years and had no known FH of CRC documented) who were seen and tested by genetics services were actually found to be carriers of Lynch syndrome, and as such fell into the high risk category. Perhaps insufficient FH documentation is responsible for these unexpected results. The findings of this study are in accordance with the published literature. A similar multi-centred English audit was conducted in 2011. Although using a different referral criteria in line with their own clinical practice, findings showed that the referral rate ranged from between only 35–55% [10]. The findings have also been echoed internationally; a Dutch study found that documentation of family history was sub-optimal, being correctly documented in only 16% of cases. 34% of patients with a complete FH recorded were referred genetics services [19]. In 2009, an Australian study showed even poorer outcomes with only 54% of patients having FH documented, and only 12% of patients being referred for formal genetic testing [20]. Another 2012 study from Australia claimed only ﻿38% of CRC patients were asked about their family by a health care provider [21]. The reason behind these findings may have been due to documentation errors; perhaps if a negative FH was found on enquiry, no FH documentation was made at all. However, even if this were the case, opportunities for patient referral to genetic services are almost certain to have been missed.

Despite meticulous data collection from an 11 year time period with at least 2 years survival follow-up, this study does have its limitations. Firstly, the cohort size of 345 patients is relatively small. When considering the division of patients into further lesser subgroups (for example, by age), this has obtained results often with a low power. Only 80.6% of patients had at least 5 years of follow-up at data collection. When the 67 individuals diagnosed after August 2013 (since data was collected in August 2018) who did not have 5 years follow-up are excluded, the values in Table 2 remain consistent. The data collection process introduced a degree of bias since most patient information was collected from oncology notes and other hospital notes including ward clerking. Pathology reports were less often accessed as this was more time consuming. Where patient notes were deemed unavailable, it was likely that these patients did not receive any oncology treatment due to early stage disease requiring only surgical intervention. Two separate researchers collated the data; one gathered data from 2005 to 2009 and the other gathered data from 2010 to 2015. Despite efforts to ensure data collection was identical both times, there may have been minor discrepancies in methods, recording and benchmarks. As the SIGN (2003) guidelines were in use during 2005–2009, they were also used as the guideline standards for the 2010–2015 cohort accepting that the SIGN guidelines were updated in December 2011. These new guidelines aimed to further improve the uptake of genetic referrals, although differences between the 2003 and 2011 standards were negligible regarding young-onset CRC and did not affect this study [13, 14]. Routine assessment for Lynch syndrome is now the standard assessment of a tumour, and patient referral is no longer required. Re-audit will allow ascertainment in change of policy. ARI’s pathology dataset was updated in July 2014 to include immunohistochemistry (IHC) analysis looking at microsatellite mismatch proficiency on all specimens from patients aged less than 50 years. Since then, in 2015, all patients having a resection for a diagnosis of colorectal cancer in the North East of Scotland have had the KRAS, BRAF and MSI status assessed on their surgical specimen. As patients with rectal cancer may require pre-operative therapy, immunohistochemistry (IHC) analysis for microsatellite proficiency is carried out on biopsy specimens, as this may influence therapeutic options. Therefore, significant improvements have been made with regard to identification of hereditary cancer syndromes.

## Conclusion

In this study, overall survival in the under 55 s was found to surpass that of CRC across all ages. This is important to discuss with yCRC patients, as perceptions in the general population reckon that young adult patients with cancer have an inferior outcome compared to patients with later-onset disease. However, patients aged under 40 years were found to have more advanced disease and a slightly poorer prognosis than those aged over 40 years. Since the Scottish Bowel Cancer Screening Programme will not detect cases in patients under 50 years, healthcare professionals should be especially vigilant. A significant lack of appropriate patient referral to genetic services was found from 2005 to 2015. Thus, opportunities for identification of potential hereditary cancer syndromes and screening are likely to have been missed in a significant high-risk patient group. Although many patients did not receive genetic testing, 18% of those who were tested were found to carry Lynch syndrome or FAP.

## Data Availability

All data generated during this study are included in this published article. The datasets used during the current study are available from the corresponding author on reasonable request.

## Abbreviations

APC: Adenomatous polyposis coli
ARI: Aberdeen Royal Infirmary
CI: Confidence interval
CRC: Colorectal cancer
FAP: Familial adenomatous polyposis
FH: Family history
IBD: Inflammatory bowel disease
IHC: Immunohistochemistry
MSI: Microsatellite instability
SD: Standard deviation
SIGN: Scottish Intercollegiate Guidelines Network
TNM: Tumour, Nodes, Metastasis
YCRC: Young-onset colorectal cancer
